# Brucellosis in the Middle East: Current situation and a pathway forward

**DOI:** 10.1371/journal.pntd.0008071

**Published:** 2020-05-21

**Authors:** Ramin Bagheri Nejad, Rosina C. Krecek, Omar H. Khalaf, Nabil Hailat, Angela M. Arenas-Gamboa

**Affiliations:** 1 Department of Microbiology and Immunology, Faculty of Veterinary Medicine, University of Tehran, Tehran, Iran; 2 Department of Bacterial Vaccines, Razi Vaccine and Serum Research Institute, Agricultural Research, Education and Extension Organization, Karaj, Iran; 3 Independent Scholar, Texas, United States of America; 4 University of Johannesburg, Department of Zoology, Auckland Park, South Africa; 5 Department of Veterinary Pathobiology, College of Veterinary Medicine & Biomedical Sciences, Texas A&M University, Texas, United States of America; 6 Department of Veterinary Pathology & Poultry Diseases, College of Veterinary Medicine, University of Baghdad, Baghdad, Iraq; 7 Pathology Laboratory, Faculty of Veterinary Medicine, Jordan University of Science and Technology, Irbid, Jordan; Faculty of Science, Ain Shams University (ASU), EGYPT

## Abstract

Brucellosis is a bacterial endemic zoonotic disease of global significance with detrimental impacts on public health and food animal production. It is caused by *Brucella* spp., an expanding group of pathogens able to infect various host species. Bovines and small ruminants, which excrete the bacteria in milk and in reproductive discharges, are major sources of infection for humans and other animals. Contact with contaminated animals and consumption of unpasteurized dairy products are the main routes for human infection. In spite of the considerable progress of knowledge gained and success achieved in brucellosis control in the developed world, this disease continues to be an important burden in the Middle East (ME). Common risk factors implicated in the difficulty and complexity of brucellosis control within the region include (1) social and political instabilities; (2) insufficient resources and infrastructure for appropriate diagnosis, reporting, and implementation of control measures; (3) variation of livestock husbandry systems and their commingling with other livestock and wildlife; and (4) traditional cultural practices, including consumption of unpasteurized dairy products. Development of core interdisciplinary competencies is required for a true One Health–based endeavor against the disease. National awareness and educational programs addressing all population sectors from consumers to decision-makers seem to be the next logical, sustainable, and economically viable approach toward improving disease status in this region. In the present review, we describe the current situation of brucellosis in the ME, focusing on the major limitations and shortcomings regarding disease control. We propose a regional approach toward public awareness of brucellosis as the first step in mitigating the disease and discuss the potential benefits, and components of such a strategy, which can further be used as a model for other endemic zoonotic diseases.

**Key learning points**

1. Brucellosis continues to be an important endemic zoonosis in the Middle East (ME) countries with significant effects on human and animal health.
2. Current situation of brucellosis in the ME region is associated with the social, political, infrastructural and resource barriers which hinder a true One Health approach to mitigate and manage control of this disease.
3. Implementation of awareness and education campaigns, targeting all stakeholders and the populace, provides a sustainable and effective pathway forward toward mitigation of the disease burden in the region.
4. For campaigns to be successful, national and local authorities of key disciplines are called upon to collaborate to establish a sustainable One Health framework.
5. Informed stakeholders are empowered to contribute to preventive interventions, assume their own share of responsibility for disease control, and engage purposely in community health.

**Top five papers**

1. Musallam II, Abo-Shehada MN, Hegazy YM, Holt HR, Guitian FJ. Systematic review of brucellosis in the Middle East: disease frequency in ruminants and humans and risk factors for human infection. Epidemiol Infect. 2016;144(4):671–685. doi: 10.1017/s0950268815002575. PubMed PMID: 26508323.
2. Godfroid J. Brucellosis in livestock and wildlife: zoonotic diseases without pandemic potential in need of innovative one health approaches. Arch Public Health. 2017;75:34. doi: 10.1186/s13690-017-0207-7. PubMed PMID: 28904791; PubMed Central PMCID: PMCPMC5592711.
3. Racloz V, Schelling E, Chitnis N, Roth F, Zinsstag J. Persistence of brucellosis in pastoral systems. Rev Sci Tech. 2013;32(1):61–70. PubMed PMID: 23837365.
4. Cleaveland S, Sharp J, Abela-Ridder B, Allan KJ, Buza J, Crump JA, et al. One Health contributions towards more effective and equitable approaches to health in low- and middle-income countries. Philos Trans R Soc Lond B Biol Sci. 2017;372(1725). doi: 10.1098/rstb.2016.0168. PubMed PMID: 28584176; PubMed Central PMCID: PMCPMC5468693.
5. Shiferaw ML, Doty JB, Maghlakelidze G, Morgan J, Khmaladze E, Parkadze O, et al. Frameworks for Preventing, Detecting, and Controlling Zoonotic Diseases. Emerging Infectious Diseases. 2017;23(13). doi: 10.3201/eid2313.170601.

## Introduction

Brucellosis is a globally important endemic zoonotic disease that is caused by gram-negative coccobacillus bacteria belonging to the genus *Brucella* [1, 2]. This genus currently consists of 12 species, but there are other potential species that might be included in the future [3]. *Brucella* spp. can infect various animal hosts as well as humans [2]. Among classical *Brucella* spp., *B*. *melitensis* and *B*. *abortus* are of paramount zoonotic importance worldwide, primarily infecting small ruminants and cattle, respectively [4]. Livestock brucellosis has detrimental socioeconomic effects in vulnerable low-income communities, particularly in the Middle East (ME) [5–8]. The disease is principally manifested with late-gestation abortion, fetal death, infertility, and reduced productivity in livestock [4]. However, the real economic losses imposed by the disease on livestock production in endemic resource-poor areas go beyond these apparent direct effects, leading to devastating impacts on socioeconomic development and the promotion of poverty [5].

Humans are considered incidental hosts that can be infected through contact with animals and animal products [1, 2]. Acute infection manifests as a disabling flu-like syndrome with nonspecific clinical signs including an undulating fever, sweating, chills, myalgia, arthralgia, and fatigue [8, 9]. If the disease is not properly diagnosed in its acute phase and is left untreated, it can become chronic and persist for years. The chronicity of infection results in localization of the bacteria in various tissues and organs causing debilitating complications such as osteoarticular, hepatobiliary, central nervous system, and cardiovascular involvement [10]. *B*. *melitensis* is the most frequently identified cause of human brucellosis in endemic regions around the globe [11] and is considered an important foodborne pathogen in developing countries. Animal-to-human transmission occurs chiefly through consumption of unpasteurized milk and dairy products from infected livestock [2], but direct contact with infected animals is considered as important as foodborne transmission in developing countries [12]. Although the disease in people is not associated with high mortality, it triggers a broad spectrum of tangible and intangible costs that affect both individuals and the community [5].

Brucellosis continues to be an important animal and public health burden in the ME [5, 7, 8], a group of nations with common geohistorical, developmental, and cultural features [8]. Similar social, educational, and health conditions prevailing in the region favor endemicity of the disease [8, 12]. Traditional customs, such as the consumption of unpasteurized dairy products and the high popularity of pastoralism, provide a means of animal-to-human transmission [13]. Additionally, social and political instability and lack of required financial and manpower resources hinder the development and implementation of a continuous program for the control of the disease [13], although considerable gains in knowledge have been achieved through decades of experience in combatting the disease in different parts of the world. It is well known that for a control program to be effective and successful, it needs a sustainable and a truly One Health approach [14, 15]. The One Health concept is based on the interdependence of human health, animal health, and environmental health and focuses on the need for cooperative decision-making, planning, and action to address health problems [16]. Such an approach requires encompassing necessary discipline-specific and interdisciplinary collaborative arrangements and activities [12, 14, 15] whose prerequisite infrastructures are not likely to be provided in the near future in the ME because they demand time, coordinated organization, and authorization [17], as well as economic resources that are currently unavailable. Therefore, national awareness and educational programs addressing all population sectors from consumers to decision-makers seem to be a next logical, sustainable, and economically viable approach to improve disease status in this region. In the present review, we describe some of the major limitations and shortcomings specifically confronted in the ME, and we propose a regional approach toward public awareness of brucellosis as the first step in controlling the disease. Finally, potential benefits and components of such an approach are discussed, which can further be used as a model for other zoonotic diseases.

## Methodology

To carry out a comprehensive literature review, internet-based databases PubMed and Google Scholar were searched for the most relevant and recent English articles before September 2018, using a combination of key words including brucellosis, Middle East, awareness and education, One Health, and control. Areas of interest included the status of brucellosis in the ME countries, risk factors associated with the disease, a One Health approach with strategies to control brucellosis in developing countries, and related awareness and educational campaigns. The most recent data on human and animal populations, land area, economic statistics, and numbers of reported brucellosis cases were also retrieved from official online databases of The World Bank, Food and Agriculture Organization of the United Nations (FAO), and World Organization for Animal Health (OIE).

## Complexity in the control of brucellosis in the ME

### Human and animal populations

The ME region consists of countries that account for about 5.3% and 5.2% of the world’s population and land area (Table 1). Around 6% of the cattle, sheep, and goats of the world are located in these countries, in which small ruminants are dominant, constituting more than 85% of the total mentioned ruminant livestock. This demonstrates the critical importance of small ruminants in the food supply in the ME countries with their considerable contribution to the provision of dairy products, red meat, and wool and hair, as documented in Iraq [18]. Sheep and goat density in this area is almost twice as much as that in the world, suggesting higher concentrations of these animals. Additionally, the actual density of small ruminants is increased in areas in which their production is confined because of substantial amounts of land with dry climate and rainfall being less than 150 mm per year, resulting in higher human–animal contact.

**Table 1:** Comparative data about human and animal populations, densities, and percentage in the ME countries.

Country	Human Population† (Thousands)	Area of Land‡ (km^2^)	Per Capita GDP§ (USD)	Cattle¶ (Head)	Sheep¶ (Head)	Goat¶ (Head)	Livestock Density (Head/km^2^)	Small Ruminant Density (Head/km^2^)	Small Ruminant Percent
**Bahrain**	1,492.58	771	23,655	10,022	38,854	17,992	86.7	73.7	85.0
**Cyprus**	1,179.55	9,240	25,233.6	67,027	311,700	254,421	68.5	61.3	89.4
**Egypt**	97,553.15	995,450	2,412.7	5,064,509	5,697,716	4,351,545	15.2	10.1	66.5
**Iran**	81,162.79	1,628,760	5,415.2	4,879,363	40,029,687	15,711,084	37.2	34.2	92.0
**Iraq**	38,274.62	434,320	5,165.7	1,899,370	6,633,904	1,282,856	22.6	18.2	80.7
**Israel**	8,712.4	21,640	40,270.3	543,311	519,640	89,720	53.3	28.2	52.9
**Jordan**	9,702.35	88,780	4,129.8	72,644	3,057,948	770,771	43.9	43.1	98.1
**Kuwait**	4,136.53	17,820	29,040.4	30,630	664,654	197,768	50.1	48.4	96.6
**Lebanon**	6,082.36	10,230	8,523.7	81,262	458,112	516,803	103.2	95.3	92.3
**Oman**	4,636.26	309,500	15,668.4	389,130	593,420	2,257,090	10.5	9.2	88.0
**Palestine**	4,684.78	6,020	3,094.7	40,254	747,880	215,000	166.6	159.9	96.0
**Qatar**	2,639.21	11,610	63,505.8	21,675	287,231	169,232	41.2	39.3	95.5
**Saudi Arabia**	32,938.21	2,149,690	20,760.9	364,958	9,328,455	3,670,440	6.2	6.0	97.3
**Syria**	18,269.87	183,630	NA^**^	1,141,833	17,641,877	2,437,521	115.6	109.3	94.6
**Turkey**	80,745.02	769,630	10,540.6	14,080,155	30,983,933	10,345,299	72.0	53.7	74.6
**United Arab Emirates**	9,400.15	83,600	40,698.8	104,584	2,208,451	2,264,699	54.8	53.5	97.7
**Yemen**	28,250.42	527,970	NA	1,696,611	9,324,541	8,944,759	37.8	34.6	91.5
**Total ME**	401,609.83	6720691	-	30487338	128,528,003	53,497,000	31.6	27.1	85.7
**World**	7,530,360.15	129,754,723.4	10,714.5	1,575,042,416	1,363,781,956	1,174,322,598	31.7	19.6	61.7
**ME Percent**	5.3	5.2	-	1.9	9.4	4.6	-	-	-

† Source: The World Bank [19].

‡ Source: The World Bank [20].

§ Source: The World Bank [21].

¶ Source: Food and Agriculture Organization [22].

^NA^ Not available.

GDP, gross domestic product; ME, Middle East.

### Current knowledge on human brucellosis

Human brucellosis is endemic in most of the ME countries with Syria, Iraq, Saudi Arabia, Turkey, and Iran having the world’s highest incidence rates [1, 9, 23, 24]. Table 2 shows recent available data on annual numbers of human brucellosis cases reported in 14 ME countries.

**Table 2:** Annual numbers of human brucellosis cases reported in some ME countries in recent years.

Country	Number of Human Cases†	Incidence Rate/100,000*
Egypt	3,756†	3.8
Iran	15,103‡	18.6
Iraq	1,004§	2.6
Israel	348§	4.0
Jordan	441‡	4.5
Kuwait	446§	10.8
Oman	416‡	9.0
Palestine	894§	19.1
Qatar	114§	4.3
Saudi Arabia	4,062‡	12.3
Syria	7,411‡	40.6
Turkey	6,457§	8.0
United Arab Emirates	47§	0.5
Yemen	25,041‡	88.6

† Latest data available on World Animal Health Information Database; †2014 ‡2016, §2017 [25].

* Using population data retrieved from The World Bank [19].

ME, Middle East.

The actual burden of human brucellosis may be far greater than the figures reported here. Data are often incomplete because they are based on national statistics and/or passively collected information from hospitals and diagnostic laboratories. This mechanism of data acquisition is excessively prone to underestimation due to misdiagnosis and reporting errors [9, 24]. For example, a rural area of Iran has demonstrated that over 40% of brucellosis patients are not reported to the highest level of surveillance authorities, which can lead to considerable variation in incidence rates across districts [26]. These differences are attributed to demographic, occupational, and socioeconomic conditions [9]. Thus, it seems that the current disease status is much more complicated, specifically in rural areas with low socioeconomic situations [27–30] and among those with high-risk occupations [31].

### Current prevalence and reporting in animals

Strategies recommended for the control and elimination of livestock brucellosis depend on current disease prevalence and reporting rates. Although valid information on exact epidemiology of animal disease is essential to making accurate decisions for adequate measures, there is little country-wide data about the real prevalence of brucellosis in ruminant livestock in the ME countries [13, 24]. However, high human incidence rates and available studies suggest its extensive presence and prevalence [7]. These limited studies are mainly based on serological surveys at local levels [7, 32–36]. Because of preferential pathogenicity of *Brucella* spp. in animal hosts, serological surveys impose limitations on the estimation of actual disease epidemiology leading to misconceptions [14] that may be due to a lack of microbiological- and molecular-based diagnostic capabilities. Currently, there is only one reference laboratory for brucellosis accredited by the OIE in the region, which is in Israel [37]. Generally, an overview of data available reveals that brucellosis in small ruminants is more prevalent than what is typically reported [24, 32–34, 38], while it might be less addressed in preventive programs [33, 39]. As *B*. *melitensis* is the most commonly identified cause of human infection, this underscores the paramount importance of small ruminant brucellosis.

### Gaps in implementation of control strategies

For successful mitigation of animal brucellosis, sustainable maintenance of a control program is pivotal [13, 40]. In recent decades, a considerable number of countries in the ME region have experienced war and social/political conflicts and have experienced large numbers of humans, animals, and animal products moving across borders. As a consequence, implementation of control measures has been repeatedly abandoned impeding effective continual results [13] which has led to disease re-emergence [13, 24]. For instance, in Yemen, the number of human brucellosis patients recorded in 2016 (25,041 cases) had doubled since 2015 (12,353 cases) [25]. Similarly, in Jordan there has been almost a five-fold increase in the number of reported human brucellosis cases from 2012 to 2016 (96 and 441 cases, respectively) [25]. This type of instability is also known to result in increased emigration of skilled human specialists and decreased available competent human resources [41]. Furthermore, reduction of unrestricted livestock transportation across borders, or illegal trade, which are common in the region [13, 32], requires expanded regional collaborations [6]. However, current political struggles between neighbors hamper necessary cooperation for sustained strict border control.

It is generally accepted that livestock brucellosis control and elimination, which is crucial for mitigation of the disease in humans, is a veterinary responsibility [42], but it is costly and requires sufficient financial resources [43]. Additionally, the per capita gross domestic product (GDP) for half of the ME countries is less than the average of the world (Table 1). Because of the nonspecific consequences of brucellosis, the real disease burden is not fully diagnosed, and therefore, investment in disease prevention is not prioritized [44, 45]. Intensive industrial herds/flocks are of significant economic importance for individual country governments to meet national animal food demand in a climate of limited resources. Consequently, preventive policies are generally focused on national animal production systems. Control programs frequently neglect animal health issues and services in households, rural areas, and pastoral systems, causing an inequity in providing adequate veterinary services such as animal identification, tracking live animal trade, quarantine, vaccine provision, and disease screening. Animal vaccination is indisputably an effective control measure that can significantly reduce the prevalence of brucellosis in livestock (and thereby, human infection), provided that well-controlled, high-quality vaccines and optimized methods are used according to the OIE standards with required coverage in the appropriate target species. Implementation of mass vaccination for brucellosis in small ruminants of many ME countries is the leading and nearly the only preventive measure in practice [24]. However, the breadth of vaccination coverage, which is essential for vaccination campaign efficiency [46], is not known. Additionally, vaccination alone cannot eliminate the disease in animal populations [47]. Even if vaccination is accompanied by other measures such as test-and-slaughter, the intensity is not enough to ensure disease mitigation [39]. It is also noteworthy that the use of low-quality vaccines can lead to a false sense of security and thus, a general unawareness of other necessary preventive practices [46]. For example, in countries such as Jordan, although sheep and goats are vaccinated with Rev-1 vaccine, use of a reduced dose of live attenuated vaccine is not uncommon, which raises questions of efficacy in these livestock species. Additionally, cattle and camels are seldom vaccinated at all.

### Epidemiological and sociocultural challenges

A factor that compounds the animal brucellosis situation in the ME is infection of different animal species as an outcome of mixed animal production practices and trans-species transmission [6, 7, 36, 48–51]. Although a thorough understanding of the epidemiology of the disease in nonpreferential host species is not yet completely understood, and these species are considered spill-over hosts [12], their likely role in pathogen maintenance and conveyance cannot be overlooked. Additionally, no reliable data are available for brucellosis in wildlife in the ME, notwithstanding its possible epidemiological effects on disease re-emergence in livestock [12, 52].

As a consequence of specific geoclimate and land conditions (aridity and altitude), traditional systems for the production of ruminant livestock (including pastoralism and nomadic practices) remain extensively common and widespread in the ME, especially for small ruminants [18, 24, 53–55]. In addition, brucellosis is enormously under-diagnosed and under-reported among mobile pastoralists because of barriers of inaccurate diagnosis of the disease and lack of surveillance systems [28, 55, 56]. Long-term sustained control of brucellosis in pastoral settings is difficult because of inaccessibility of competent public and veterinary health services, close contact between animals and their owners, ingestion of unpasteurized dairy products, and seasonal changes in livestock composition [28, 55, 56]. Owing to the complete economic and cultural dependence of pastoral communities on their livestock [55, 57], implementing strategies based on culling infected animals is not acceptable, because animals are often the primary source of livelihoods. Furthermore, the mobile nature of pastoral livestock production is detrimental to the control of animal movement, which is required for disease control. Therefore, the disease has a stable transmission level and tends toward persistence and endemic stability [55]. Despite scarcity of comprehensive information on disease status, several studies reveal high prevalence and incidence of brucellosis in nomadic pastoral communities [27, 28, 40, 55, 56, 58, 59].

The magnitude of *Brucella* infection in humans depends upon factors such as dietary habits, methods of processing milk and milk products, husbandry practices, and environmental hygiene [13], which are all associated with awareness of the disease amongst consumers and producers. A number of knowledge, attitudes, and practices (KAP) as well as risk factor analysis studies in the region show low levels of awareness and popularity of risky behaviors/practices among people, especially livestock producers [29, 38, 40, 51, 60]. Remarkably, even with knowledge about disease aspects such as presence, symptoms and routes of transmission, risky behaviors persist [38, 51, 60]. The situation is more serious in rural areas among householders and pastoral livestock producers as reported in other countries [29, 61–63]. Risky practices widely reported include consumption of unpasteurized dairy products (e.g., fresh cheese and raw milk) [35, 38, 51, 64], as many livestock producers disbelieve this mode of transmission and risky practice, and unprotected handling of animals and animal remains [31, 38, 51, 60]. Existence and popularity of unsupervised traditional dairy markets further exacerbate the situation [18]. Furthermore, many pastoral livestock producers believe that vaccination against brucellosis may harm their animals and cause abortions and refuse to vaccinate their flocks, further complicating any control program for brucellosis.

## Awareness programs as the next logical approach

### Why awareness programs?

**Fig 1:**
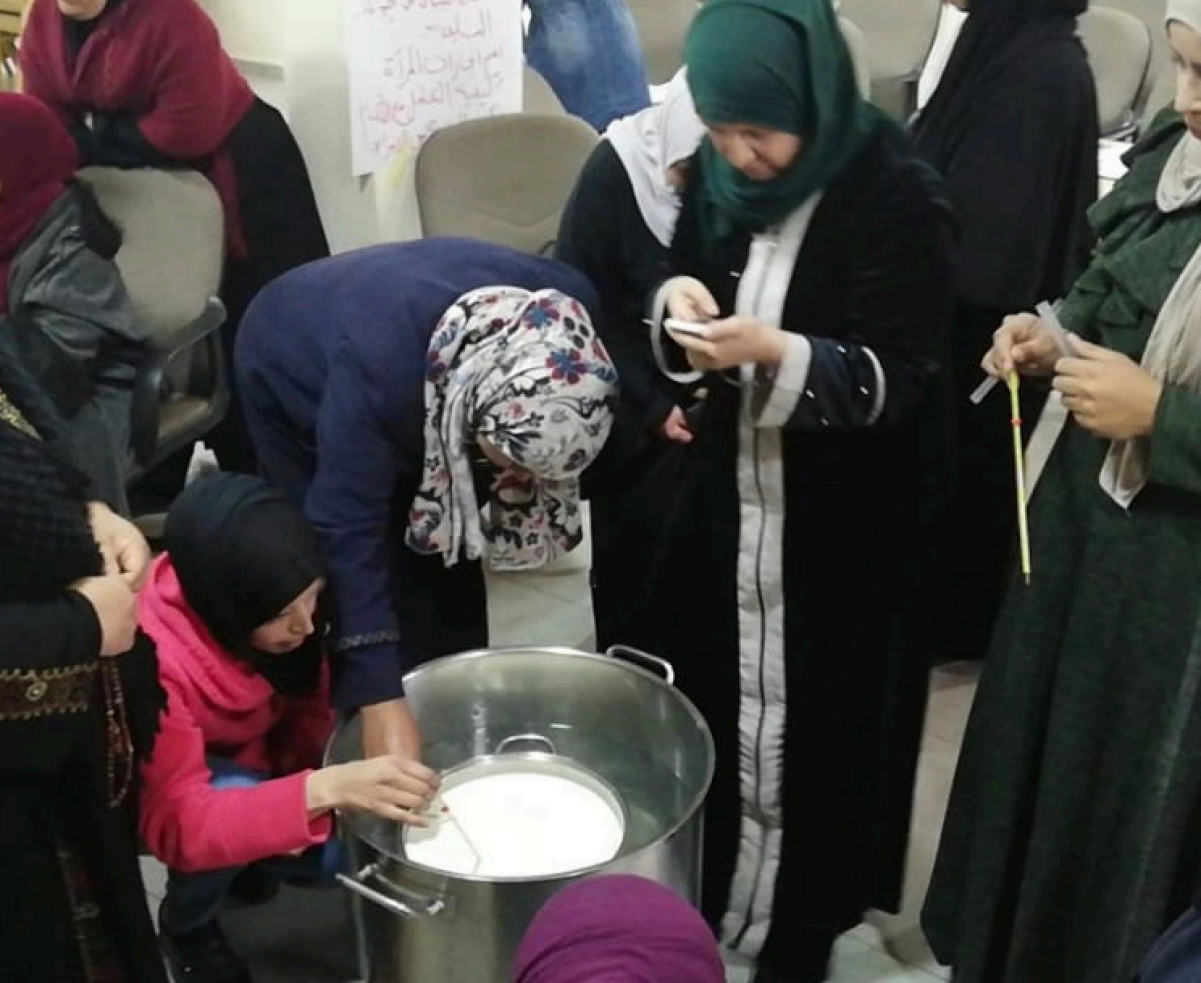
Locals receiving training on how to pasteurize milk.

As with many other zoonoses, the main reason for the "neglected" state of brucellosis is not the lack of knowledge about the disease epidemiology, the causative agent, or effective control methods. Rather, it is because of a lack of actual disease burden data in vulnerable, impoverished sections of low- and middle-income populations and the little political and social influence this group of people has on decision-making [44, 45, 65]. Therefore, to reduce disease neglect, it is necessary to circumvent disregard of brucellosis-prone communities, which can also lead to distrust of official organizations, policies, and measures [66]. Because nonspecific symptoms of brucellosis (e.g., fever in humans and abortion in livestock) may also be present following successful implementation of control measures, benefits of disease control are indiscernible. As a result, stakeholders and communities can contribute to a "neglected" state of the disease by inadequate appreciation of its importance and denial of disease control benefits, which can impact stakeholders’ contributions to control programs [44, 67]. These issues can be addressed by development and implementation of regionally oriented national awareness programs. Additionally, quality education is a principal human right recognized by the United Nations [68], which enables people to improve their living conditions and is a critical contribution to socioeconomic development alleviating poverty and social inequalities [69]. Equitable education and awareness programs diminish the impact of poverty and sociocultural factors on people at an individual level through appropriate health-associated behaviors and mitigation of disease risks [44]. This approach contributes to a One Health intervention for high-risk populations (pastoralists and rural households) and is considered a first practical and economic step toward inequity alleviation in preventive care measures (Fig 1).

### Multidisciplinary and multileveled involvement in awareness programs

The current complicated status of brucellosis across endemic countries in the ME can be strategically tackled with the realization of the importance of prioritization, investment, policy-making, legislation, and both financial and official facilitation of multidisciplinary actions. To achieve this goal, appropriate intersectoral cooperation and multidisciplinary involvement should be realized. Along with public health and veterinary authorities, other governmental administrative bodies are required to be involved in the planning and execution of these programs, which are responsible for social welfare, rural and pastoralists' affairs, basic and higher education, environment, national and local financing, and municipalities [66]. Awareness programs should raise the visibility of disease impact to all stakeholders, including legislators, national and local responsible policy-making authorities, veterinary practitioners and workers, physicians and healthcare providers, livestock producers, dairy product processors and vendors, remote nomadic and rural smallholder farmers, and customers, who all have significant roles in disease prevention and provide overarching links between all sectors [44, 65].

### Urging political decision-makers

To increase regional and national awareness, political decision-makers are critical to gaining approval from legislative institutions to promote transboundary cooperation. They are also critical to driving allocation of required funds and resources. Therefore, a comprehensible conceptual framework with essential requirements, including a holistic multidisciplinary One Health approach, is urgent [5, 65]. This should include a clear understanding of the brucellosis burden and its impact, as well as the public health, economic, and social goals required to control and mitigate this disease [65]. Decision-makers need to also be informed of the importance of Biosafety Level 3 diagnostics and vaccine quality control laboratories, as well as novel microbiological and molecular methods for effective disease control [45, 70]. National reference laboratories with well-educated, skilled personnel capable of bacterial isolation, identification, and strain characterization for molecular epidemiology can play a central role in national surveillance systems. These laboratories should collaborate with both public health and veterinary field sectors to perform routine serological and microbiological surveys, which provide sound country-wide epidemiological data for evidence-based decision-making. To convince political decision-makers of the urgency for adequate financial allocation, awareness of the need for sufficient investments for multidisciplinary capacity-building and facility/organizational/manpower infrastructures for brucellosis control programs is required. This capacity and infrastructure platform can also be applied to coordinated actions against other endemic zoonotic disease risks, which can avoid parallel expenditures [44]. Regular meetings of professional bodies, including distinguished specialists and senior politicians, to share information of the economic and health losses caused by brucellosis at national and local levels can prove to be invaluable. Experts with appropriate experience with this disease, its mitigation, and the One Health approach in the developing world should be invited to contribute to these meetings. In addition, politicians should be invited to participate from the region and international bodies (e.g., The World Bank, OIE, FAO, etc.). Together, this political decision-maker team should focus on how to build cooperation with country neighbors, mitigate illegal and uncontrolled animal movement across borders and animal/animal products trafficking, employ quarantine at borders that conform to the OIE standards for animal/animal product trade, and underscore the importance of technical and scientific collaborations with international human and animal health authorities. International authorities such as WHO, OIE, and FAO have the power to facilitate negotiations and agreements at a political level between opposing countries in a region, thus promoting regional harmony. These organizations can further educate public health and veterinary policy-makers about the most recent scientific knowledge on control and elimination strategies, including optimized use of available vaccines in target animal hosts, vaccine quality standards, and other interventions that will allow them to make the most appropriate policies according to their disease epidemiological situation and available resources in ME countries.

### Public education and awareness

Widespread education and awareness programs applying a One Health approach have demonstrated effective and equitable results to animal and human health in developing countries [44, 67]. Communicating the threats of disease and solutions to the public is essential to shaping public decisions and behaviors and is necessary for the prevention of disease and health improvement [71]. Informing stakeholders (e.g., consumers, livestock farmers, producers and at-risk populations, policy-makers, disease experts, One Health experts, neglected tropical disease specialists, etc.) of the disease and associated high-risk behaviors will embolden them to appreciate the importance of public education and awareness internationally and regionally.

Public education and awareness can lead to reduction of disease transmission from animals to humans [5], which is an effective and economically viable approach in the front line of disease management [45]. Also, public education is considered the most sustainable method to raise community trust of authorities and identify interventions together through regular interactions of stakeholders. The goal is to enhance societal engagement of a disease control program by provoking a public, regional, and global sense of ownership [44].

Health communication methods are used to raise public awareness of disease and different facets of mitigation [71]. For example, social marketing campaigns for dairy food production and consumption practices using commercial multimedia tools such as flyers, leaflets, billboards, newspapers, and television shows in local languages are popular and feasible [18, 71]. The audience, the message, the sources, and the channels used for communication are crucial factors that should be considered when planning such campaigns [71]. Messages should be culturally sound, relevant, and simple to understand [18, 71]. As the process of integration of messages into people's lives takes time, programs should be adjusted to the stage of the campaign and pursued until an effective change is made. Apart from being a warning in nature, messages should provide data on losses associated with the disease and benefits of prevention in order to make these aspects visible.

Messages should be presented when and where there are risks of disease transmission. For instance, large pictorial posters and infographics can be used in traditional dairy markets and shops to advocate a message about avoiding consumption of raw milk in areas where people believe pasteurization and heating of milk destroys its nutritive ingredients. Public transportation vehicles (buses and trains) are examples of places where health messages can be delivered to the audience publicly. Mass media information (especially by television) can help to change public food consumption habits. Culturally accepted entertaining television movies and shows can also be used [71]. Interviews with well-known specialists and eminent figures warning of dangers caused by brucellosis and providing information on ways of prevention can have significant impact. Sometimes it may be useful to stimulate public emotions with real-life stories from infected people explaining how they suffered [71]. Popularity of mobile phones provides a powerful means of reaching people (e.g., text messaging and WhatsApp). The application of a One Health model was introduced in educational programs for women in a brucellosis endemic region of Iran, which resulted in a significant decrease in KAP scores 1 month after short-term education courses [72]. However, in contrast, a short-term awareness campaign for rabies in Azerbaijan was not successful in demonstrating change in postexposure health-seeking behaviors [73]. These opposing results of 2 different diseases suggest that sustained long-term planning is required for a comprehensive One Health awareness and education campaign to be successful in modifying behaviors and practices. Schools are a key means of public education to provide a sustainable and accepted way of training for future society actors. Special health and hygiene subjects regarding brucellosis should be included in schools' teaching curricula, provided that they are selected based on local needs and concerns and that they are culturally acceptable. Moreover, adolescents and youth, who are targeted at schools, have an undeniable influence on families and friends through interpersonal interactions and have great potential for promoting changes in traditional beliefs.

### Education of pastoral and rural communities

In resource-limited settings of pastoral communities and rural regions, cost-beneficial preventive interventions are needed [74]. Education is undoubtedly an example of such interventions. In addition, education is an important part of fundamental efforts for community-based surveillance by outreach to vulnerable community members who can serve as the first line of surveillance for the health of their own animals [5, 74]. For pastoralists, there are numerous possibilities of education and examples, including mobile facilities, open and distance learning, and field centers [69]. Organizing specific education sessions is a practical method [44]. Local community leaders should be involved in developing culturally and economically effective tools, techniques, and content [5]. These educational sessions should be well-designed and easy-to-understand [67, 75]. Because of the high illiteracy rates among many rural communities, educational content should be presented using multimedia implements [18]. Special considerations should be considered regarding education of women in pastoral and rural areas because women are generally more vulnerable but also can have significant positive impacts on their whole households [56]. In a 1-year program of empowering Bedouin women in the southern part of Jordan on early detection of animal disease and prevention of zoonotic diseases including brucellosis, a substantial change in KAP was found in these women toward protecting their families and their communities from such diseases.

Participation of local veterinary workers along with public health providers is essential [18], as positive perception of traditional animal owners in rural districts about integrated public health and veterinary participation in education has been reported [40, 59]. Improvement in general perception of different components of control programs such as proper vaccination, cleaning and disinfection, proper handling of potential infectious materials, culling infected animals, selecting replacement animals from brucellosis-free herds/flocks, simple and applied biosecurity measures, control of animal movements, use of personal protective equipment during high-risk practices (e.g., handling animals during parturition), and their benefits should be the aim [5, 44, 67]. Training of how to use modern mobile technologies such as smart phones for disease surveillance and information delivery can empower communities to report real-time disease events locally concerning their animals and generate a sense of ownership regarding their health, livelihoods, and welfare [67, 70, 74, 75]. Encouraging pastoral and rural people to attend educational sessions is crucial to a One Health approach [58]. These sessions may include vaccination to mitigate disease in animals and humans, anthelmintic drug delivery, performing free check-ups or screening tests for well-appreciated health problems (e.g., blood glucose or blood pressure examinations) can be offered simultaneously [74]. However, there may also be limited connection between awareness and behaviors/practices suggestive of restricted knowledge and false perceptions about risks of the disease [76]. Interaction between health providers and communities through regular education sessions provides an opportunity to identify the needs and gaps to lead to better prevention campaigns through an enhanced understanding of risks and objections [17]. Socioeconomical concerns and expectations of communities should also be considered when health issues are discussed with health professionals and trainers. However, KAP studies in each region and population help to provide information gaps and prevalent risks for targeted educational materials [67].

### Awareness among professional stakeholders

Special attention should be given to dairy product value chain actors, who may not currently be aware of biosecurity and food safety measures. Food producers including dairy processors and producers need to take responsibility for food safety. This can be addressed by creating awareness of the disease and training on how to protect these food producers as well as their customers [77, 78]. Enhancing dairy producers’, processors’ and vendors’ awareness of how increased quality results in higher prices for their products can increase their economic incentives to adhere to hygienic standards [70].

There is an urgency for adequately educated and skilled professionals and workforces who are aware of all disease aspects to gain an understanding of the One Health approach, the application of sanitary standards, risk assessment, and the necessity of integrated cross-disciplinary academic training of human and animal health–associated professionals [17, 70, 79]. Public health and veterinary academic centers also have the potential to participate in awareness and education campaigns by recruiting volunteer students and conducting epidemiological studies and data acquisition on disease prevalence and risk factors. Collaboration with reputable international academia for promotion and practice of One Health at higher education centers in the ME countries and funds spent on this issue can be reciprocally productive and beneficial [80], because a considerable number of human infections detected in the developed world are traced back to this region [11, 81].

## Conclusions

Brucellosis remains an important, regionally widespread zoonosis with extensive impact throughout the ME, and its control is complicated and hindered by sociopolitical barriers. To take a major step forward toward mitigation of the disease burden in the region, the development, implementation, and adoption of awareness and education campaigns supported by a One Health approach is urgent and potentially can make an effective change in current disease situations. These campaigns should target all influential stakeholders (e.g., politicians, authorities, professionals, producers, public, policy-makers, etc.) and engage various administrative bodies (e.g., public health, veterinary, social welfare, rural affairs, education, environment, municipal and financing authorities) and be relevant to national, local, cultural, and socioeconomic conditions. Sustained programs at the populace level to enhance public awareness and knowledge in forms of educational sessions, mass media communication, professional trainings, school teaching lessons and so forth help to enable people to take care of their own and their animals’ health, to engage in community health and welfare, and to trust officials and interventions. Consequently, stakeholders and the public, who are the main target of control measures, take over their own share of responsibility for the mitigation of brucellosis burden.
